# Symptomatic Secondary Selective IgM Immunodeficiency in Adult Man with Undiagnosed Celiac Disease

**DOI:** 10.1155/2012/684247

**Published:** 2012-11-27

**Authors:** Eli Magen, Viktor Feldman, Mishal Joseph, Hadari Israel

**Affiliations:** ^1^Leumit Health Services, Ashkelon, Israel; ^2^Medicine B Department, Barzilai Medical Center, 78306 Ashkelon, Israel; ^3^Allergy and Clinical Immunology Unit, Barzilai Medical Center, Barzilai Hospital, Ben Gurion University of Negev, Ashkelon, Israel; ^4^Orthopedic Department, Meir Medical Center, Kfar Saba, Israel

## Abstract

Selective IgM immunodeficiency (SIgMID) is a heterogeneous disorder with no known genetic background and may occur as a primary or a secondary condition. Celiac disease has been reported in association with several humeral immunodeficiencies, including isolated severe selective IgA deficiency, panhypogammaglobulinemia, and isolated combined IgA and IgM deficiency. There are only few reported cases of pediatric and adult patients with SIgMID and celiac disease. In this paper, we describe an adult patient with a symptomatic secondary SIgMID associated with undiagnosed celiac disease, with a resolution of clinical symptoms of immunodeficiency and serum IgM normalization following a gluten-free diet.

## 1. Introduction

Selective IgM immunodeficiency is a heterogeneous disorder with no known genetic background and may occur as a primary or a secondary condition, with a reported prevalence of 0.03% to 3% [1] Secondary SIgMID is often associated with several neoplasms or autoimmune diseases [2–5]. Primary SIgMID can be asymptomatic or present with a variety of bacterial and viral infections in the pediatric and adult populations [3].

In this paper, we describe an adult patient with a symptomatic secondary SIgMID associated with undiagnosed celiac disease, with a resolution of clinical symptoms of immunodeficiency and serum IgM normalization following a gluten-free diet.

## 2. Case Report

A 42-year-old previously healthy man, emigrant from Russia, with an unremarkable clinical history presented in April 2011 at our Clinical Immunology Unit with predominant symptoms of fatigue for the past 3 years. He does not smoke and consume alcohol on occasion. A review of systems was significant for chronic fatigue, without deterioration in short term memory or concentration, without sleep disturbances, without weight loss or fever.

Although he was able to continue working, his severe fatigue necessitated frequent time off from work, and eventually he had reduced his work commitment to part time. In September 2009, the patient was admitted into a hospital in Moscow for upper-right lobe pneumonia, and after that he frequently caught colds during 2008-2009. In 2010, the patient was admitted for pneumonia twice. These repeated episodes occurred in different lung fields. No microbiologic source of recurrent pneumonia was identified. Additionally, during 2009-2010 the patient suffered from several episodes of *staphylococcus aureus* associated skin infections.

At presentation in our clinic (January 2011), physical examination was unremarkable. After a systematic evaluation of the patient's history and complaints, comprehensive laboratory work-up of immunodeficiency was performed (Table 1). The blood cell count, biochemistry, liver enzymes, serum iron, ferritin, vitamin B12, zinc, folic acid, TSH, free T4, cortisol, T cells, T cell subsets, B cells, and natural killer cells were within normal limits. Lymphocyte transformation of phytohemagglutinin (PHA), concanavalin A (Con A), mumps antigen, and purified protein derivative antigen was normal. In vitro lymphocyte proliferative response to *Candida albicans* and tetanus toxoid antigens were also unaffected. Mantoux test was negative. Phagocytic function test using nitroblue tetrazolium and Toll-like receptor 2 (TLR2) on monocytes were normal. The patient had normal quantitative serum IgA, IgG, and IgG subclasses and IgE. However, serum IgM levels were low at 9 mg/dL. The patient was capable of normal antibody responses to pneumococcal polysaccharide antigens following Pneumovax vaccination.

Since antinuclear antibodies were positive, most clinically relevant autoantibodies were checked, but all of them were found to be negative (Table 1).

Serum ANA, anti dsDNA and anti Smith antibodies levels were measured by QUANTA Lite ELISA (Inova, San Diego, CA, USA) using the manufacturer's suggested cut-off of >20 units to define positive results for ANA and anti dsDNA antibodies, >25 IU/mL for anti Smith antibodies. Serologic evaluations of our patients were performed according to the guidelines of European Society for Pediatric Gastroenterology, Hepatology, and Nutrition for the diagnosis of Celiac Disease [6]. According to the guidelines the initial test should be IgA class anti-TG2 from a blood sample. In subjects with either primary or secondary humoral IgA deficiency, at least 1 additional test measuring IgG class CD-specific antibodies should be done (IgG anti-TG2, IgG anti-DGP or IgG EMA, or blended kits for both IgA and IgG antibodies). In our patient the total IgA level was within the normal range. The guidelines recommend tests measuring antibodies against DGP as additional tests in patients who are negative for other CD-specific antibodies but in whom clinical symptoms raise a strong suspicion of CD, but in our patient we had no clinical suspicion for CD until his son had been diagnosed. The guidelines recommend not to perform tests for the detection of IgG or IgA antibodies against native gliadin peptides (conventional gliadin antibody test) for CD diagnosis, so we did not tested the patient for anti gliadin antibodies. It should be mentioned that no genetic testing was done and the HLA type of the patient as well the parents of the patient are not suffering from CD.

Once the diagnosis of symptomatic SIgMID was recognized, the patient was treated aggressively with the courses of several antibiotics using Cephalexin, amoxicillin/clavulanic acid, and trimethoprim/sulfamethoxazole during the five additional episodes of sinusitis and skin infections throughout 13 months.

In March 2011, during the follow-up visit the patient reported that his 7-year-old son underwent colonoscopy with biopsy due to losing weight and iron deficiency, and he had been diagnosed with celiac disease. Consequently, we decided to exclude celiac disease in our patient. Repeated serum immunoglobulin A-class tissue transglutaminase antibodies were within a normal level. The patient was referred to upper endoscopy, and the duodenal biopsy samples showed villous atrophy with crypt hyperplasia. Subsequently, a gluten-free diet was recommenced. Within 4 months, chronic fatigue disappeared and no skin or sinopulmonary infections were observed during the subsequent 9 months of followup. Moreover, repeated laboratory analyses showed normalization of serum IgM levels (79 mg/dL). In February 2012, the follow-up small bowel mucosal biopsy specimen was normal; the patient was feeling well, there were no signs of any infectious disease and serum IgM level was 128 mg/dL.

## 3. Discussion

Our patient, who was previously healthy, represents a possible case of celiac disease associated secondary selective IgM deficiency. The main complains of the patient were associated with chronic fatigue and recurrent skin and sinopulmonary infections, without gastrointestinal complains or symptoms. The diagnosis of celiac disease in his son was the main reason for which we performed upper gastrointestinal endoscopy and duodenal biopsy.

Duodenal villous atrophy is not unique for celiac disease and may be observed in several immunodeficiency conditions. This pathology is frequent in symptomatic common variable immunodeficiency (CVID) [6], but our patient did not have clinical and laboratory criteria of CVID. Although the most common pathologic finding in the small intestine in CVID is villous flattening that grossly resembles celiac disease, there are some important differences between the villous flattening of CVID and celiac disease. The absence of plasma cells and the presence of polymorphonuclear infiltrate (PMNI) as well as graft-versus-host disease-like lesions (GVHDL) are thought to be pathologic features that differentiate CVID from untreated CD [7]. In CVID, the villous atrophy is thought to be T-cell mediated, while in celiac disease, there are plasma cell infiltrates with increased amounts of IgM and IgA. Moreover, in celiac disease, removal of gluten from the diet almost always leads to recovery of normal villous architecture. But, in CVID, removal of gluten from the diet improves villous flattening in only approximately 50% of patients [8, 9].

Celiac disease has been reported in association with several humoral immunodeficiencies, including isolated severe selective IgA deficiency [7, 10], panhypogammaglobulinemia, and isolated combined IgA and IgM deficiency [10]. Review of the literature revealed a few reported cases of pediatric and adult patients with SIgMID and celiac disease [3, 7, 10–12] while all reported cases were without unusual risk of infection and in all patients IgM levels returned to normal levels following a gluten-free diet [3, 10, 12] Therefore, our case is the first report of symptomatic secondary SIgMID associated with celiac disease.

The case presented here is in accordance with the previous observations that pediatric CD is very different from adult CD. In children, the classic forms are predominate and usually have positive serology and duodenal biopsies. In contrast, in adults the atypical forms predominate, with fewer positive serology, characterized by common extra-digestive complaints and various accompanying conditions, which makes diagnosis more challenging and greatly accounts for the longer diagnostic delay seen in adults [6, 13].

The pathogenesis of secondary SIgMID associated with celiac disease is unknown. It has been suggested that this secondary SIgMID is related to reduced IgM synthesis due to lymphoreticular dysfunction stimulated by gluten antigen exposure [11, 12] or/and to the defect in B cell differentiation into IgM-immunoglobulin secreting cells [14].

The problem of negative serology in untreated CD patients is becoming increasingly recognized [15]. The common reason of such seronegativity is selective IgA deficiency which may coexist with CD (roughly 2% vs. 0.2% in non-celiac controls) [16]. The reported pooled sensitivities of tTG-IgA in adults and children was 90% and 93% and endomyseal antibodies (IgA-EMA) to monkey esophagus by indirect fluorescent assay (IFA) was close to 96% and 97% in children and adults, respectively [17]. IgG anti-gliadin antibodies (AGA) testing remained the standard diagnostic test for CD in individuals with selective IgA deficiency until recently, but in the past few years, IgG anti-endomysial antibody (EMA), and anti-deamidated gliadin peptide (anti-DGP) assays have been developed that are superior to IgG AGA for this population [17]. Moreover there has been an ongoing discussion on whether AGA-positive individuals without CD (i.e., with negative antibodies against tTG and/or EMA and normal intestinal histology) may represent the mild end of the “gluten sensitivity” spectrum [18].

In conclusion, this case report should alert clinicians to the possibility that celiac disease can be associated with a symptomatic secondary SIgMID. Although rare, this condition may be associated with recurrent respiratory infections.

## Figures and Tables

**Table 1:** Immunologic studies.

	Result	Reference range
Innate immunity		

White blood cell count, 10^3^/*μ*L	6.9	4.5–10.8
Hemoglobin, g/dL	14.7	13.5–16.9
Absolute neutrophil count, 10^3^/*μ*L	4.3	2.0–8.1
Absolute lymphocyte count, 10^3^/*μ*L	1.8	0.9–3.3
Absolute monocyte count, 10^3^/*μ*L	0.6	0.0–0.8
Absolute eosinophil count, 10^3^/*μ*L	0.2	0.0–0.54
Platelets, 10^3^/*μ*L	184	150–450
C3, mg/dL	116	90–180
C4, mg/dL	29	10–40
CH50 U/mL	240	101–300
C reactive protein, mg/dL	0.06	0–5
Phagocytosis assay (% phagocytosis)	34.3	25–45

Adaptive immunity		

Lymphocyte subsets, N/*μ*L* (%) *		
CD3 + T cells	1349 (75)	620–1850 (62–84)
CD3 + CD4 + T cells	1104 (61)	345–1200 (31–61)
CD3 + CD8 + T cells	257 (14)	85–730 (10–38)
CD4/CD8 ratio	4.3	0.9–1.9
CD3 − CD19 + B cells	329 (18)	50–480 (5–26)
CD3 − CD56 + NK cells	134 (7)	15–350 (1–17)
Serum immunoglobulins		
IgM, mg/dL	9	40–230
IgA, mg/dL	172	70–400
IgG, mg/dL	1186	700–1600
IgG1, mg/dL	636	365–941
IgG2, mg/dL	345	165–545
IgG3, mg/dL	52	32–116
IgG4, mg/dL	64	6–121
IgE, mg/dL	11	0–87

Autoantibodies		

ANA	Positive	Negative
Anti-dsDNA antibody	4	Positive: >20
Anti-Smith Ab	11.5	Negative: <15 Positive: >25
ANCA	Negative	Negative
RF	Negative	Negative
IgA-tTG (EU/mL)	14	Negative: <20 Positive: >25
Mitochondrial Ab	Negative	Negative

Serological blood tests		

HIV 1,2 Ab, ELISA	Negative	Negative
HBs-Ag	Negative	Negative
HCV	Negative	Negative
TPHA	Negative	Negative
CMV Ab IgM (AU/mL)	0.13	Nonreactive: <6
CMV Ab IgG (AU/mL)	>250	Positive: >20
EBV EBNA Ab IgG	486	Reactive: >6

Abbreviations: ANA: antinuclear antibodies; ANCA: antineutrophil cytoplasmic antibodies; dsDNA: double-stranded DNA; ELISA: enzyme-linked immunosorbent assay; HIV: human immunodeficiency virus; Ig: immunoglobulin; RF: rheumatoid factor, HCV: hepatitis C Virus; TPHA: treponema pallidum particle agglutination assay; IgA-tTG: IgA anti tissue transglutaminase antibodies; CMV: cytomegalovirus; EBV: epstein barr virus; cpm: counts per minute.
